# Supplementary material for: Prediction of unobserved bifurcation by unsupervised extraction of slowly time-varying system parameter dynamics from time series using reservoir computing
Source: Front Artif Intell. 2024 Oct 22;7:1451926. doi: 10.3389/frai.2024.1451926 (PMC11534796; doi:10.3389/frai.2024.1451926)
Supplement: Supplementary file 1 [file Data_Sheet_1.pdf]

Supplementary Material

1 SUPPLEMENTARY TABLES AND FIGURES

Figure S1 shows the slow reservoir's output, $\tilde{u}^s(n)$ and the smoothed slow reservoir's output filtered by linear dynamics, $h(n)$. The filtering procedure did not affect the waveform of $h(n)$.

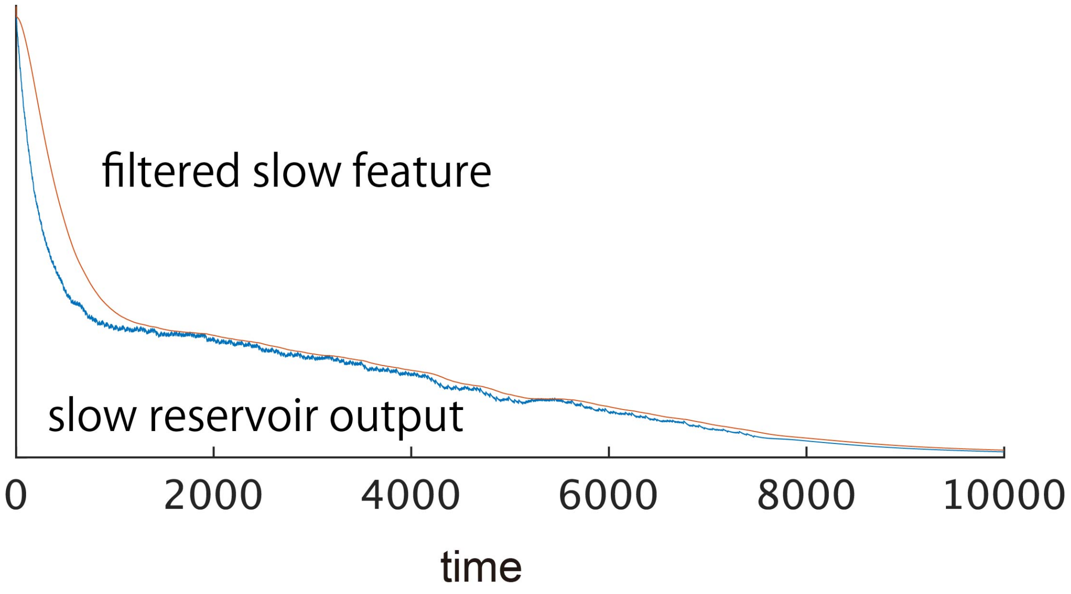

Figure S1. The slow reservoir's output and smoothed input to the downstream reservoirs. The application of a linear filter does not significantly alter the shape of the time series; it is used solely for removing high-frequency components and smoothing.

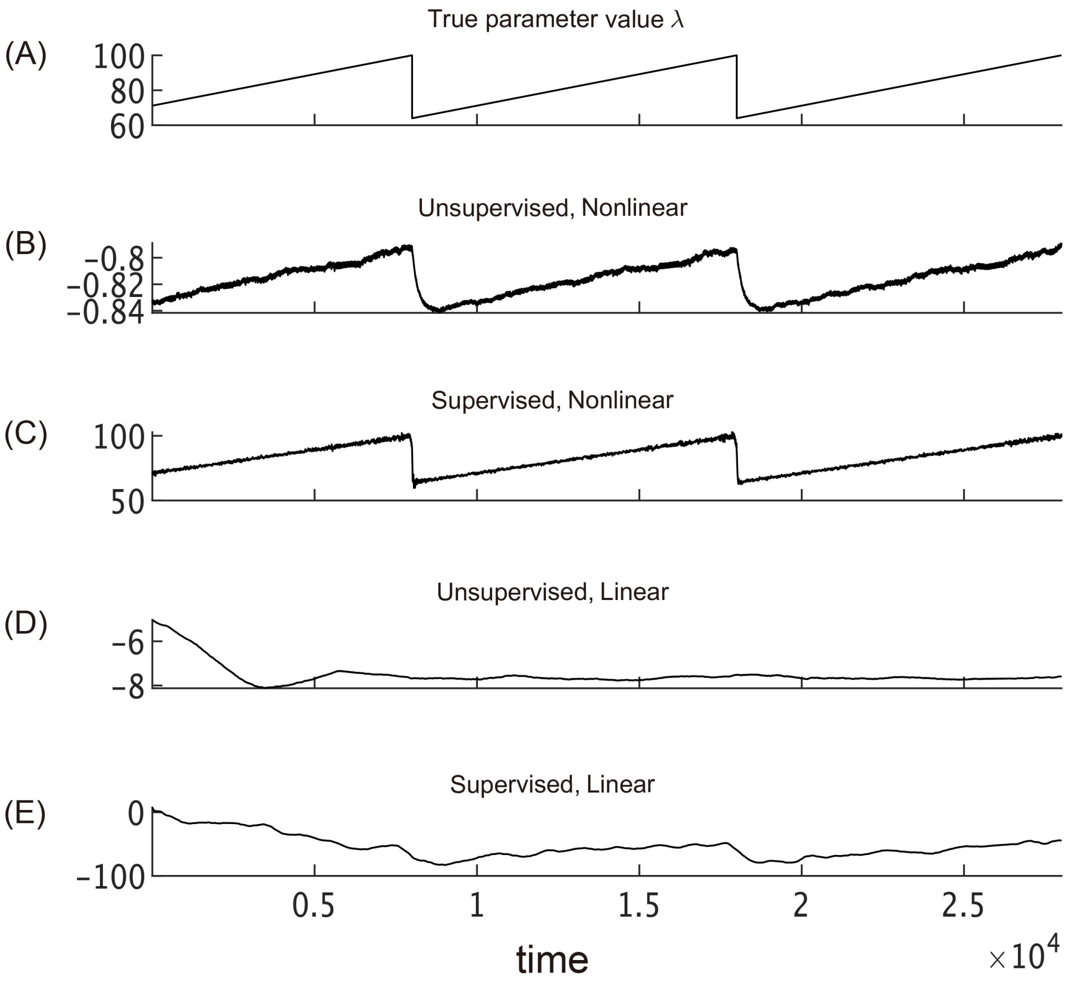

Figure S2. The slow reservoir's output and smoothed input to the downstream reservoirs. The application of a linear filter does not significantly alter the shape of the time series; it is used solely for removing high-frequency components and smoothing.

To investigate whether nonlinearity, as well as the slow timescales of the reservoir, is crucial for the extraction of slow features, we conducted identical computations using a linear reservoir in which the activation function $\tanh$ in equation 8 in the main text is replaced with the identity map (fig. S2). Figure S2(B) shows the results of slow feature extraction by the nonlinear slow reservoir, which follows a pattern similar to the true parameter variations depicted in fig. S2(A), which is the same result as in the main text. In contrast, when the reservoir's activation function $\tanh$ is replaced with an identity map, the outcomes, as shown in fig. S2(D), do not correlate with the true parameter variations shown in fig. S2(A). Furthermore, to more directly verify whether the internal states of the reservoir has information about the slowly varying true parameter values, we fitted the internal states of the reservoir to the true parameter values λ shown in fig. S2(A). Namely, the common reservoir computing framework setting for readout fitting is done. The results, depicted in fig. S2(C), reveal that fitting yields small residuals between the result and the true parameter value. However, with the linear model, even when fitting is performed in a supervised manner, fails to replicate the changes in the parameter λ (fig. S2 (E)). Therefore, the results obtained suggest that the nonlinearity of the slow reservoir is necessary.
